# Electrochemical Properties of PANI as Single Electrode of Electrochemical Capacitors in Acid Electrolytes

**DOI:** 10.1155/2013/940153

**Published:** 2013-10-20

**Authors:** Haihua Zhu, Shunjin Peng, Weijie Jiang

**Affiliations:** ^1^School of Materials and Metallurgy, Wuhan University of Science and Technology, Wuhan 430081, China; ^2^School of Science, Wuhan University of Science and Technology, Wuhan 430081, China

## Abstract

The polyaniline (PANI) powder with globular sponge-like morphology was prepared by chemical solution polymerization, and its morphology and chemical structure were characterized by scanning electron microscope (SEM) and Fourier transform infrared spectroscopy (FTIR), respectively. The single electrode of electrochemical capacitor was made using the prepared PANI powder as active material and carbon paper as current collector. Electrochemical properties of PANI as a single electrode in 1 M HCl and 1 M H_2_SO_4_ electrolyte solution were tested by galvanostatic charge/discharge (GCD) and cyclic voltammetry (CV) techniques. It has been found that PANI has higher specific capacitance of 302.43 Fg^−1^, higher specific energy of 54.44 Wh*·*kg^−1^ at 0.5 Ag^−1^, and higher working potential in 1 M HCl than those in 1 M H_2_SO_4_.

## 1. Introduction

PANI as a conducting organic material has attracted great attentions of researchers due to its good environmental stability [1, 2], moderately high conductivity upon doping with simple protonic acid [3], lower production cost, and easy synthesis compared with other conducting polymers [4], such as polypyrrole and polythiophene. Polyaniline is considered as an air-stable organic conducting polymer with interesting electrochemical performance which makes it suitable for many practical applications [5–7], especially in lithium ion batteries and electrochemical capacitor area [8–11]. Electrochemical capacitor is usually well known as a novel energy storing system, and it is quite promising in electronic area due to its excellent properties compared with batteries and traditional static capacitors, such as high power density and energy density. Their power density could be 10 times higher than ordinary batteries, and energy density could be dozens of times higher than that of traditional capacitors [12].

Electrolyte, as an important component of electrochemical capacitor, has great influence on the electrochemical properties of electrochemical capacitors [13–23]. A great number of works studied the influence of supporting acid electrolytes on PANI from electrochemical polymerization as a single electrode of the electrochemical capacitor in past years [17, 24], while there is few work for the PANI from chemical polymerization.

Therefore, in this research, we prepared globular sponge-like PANI grain by chemical methods and studied the electrochemical behaviors of PANI powder as a single electrode of electrochemical capacitor in 1 M HCl and 1 M H_2_SO_4_.

## 2. Experimental

### 2.1. Materials

Ammonium persulfate (APS, (NH_4_)_2_S_2_O_8_), HCl, H_2_SO_4_, ethyl alcohol, and aniline were purchased from Sinopharm Chemical Reagent Corporation (Beijing, China); Polytetrafluoroethylene emulsion (PTFE, 60%) and acetylene black were purchased from MTI Corporation (Hefei, China). All the chemical reagents above are in analytical grade. All the chemicals were used as received.

Fourier transform infrared spectrometer (FTIR, VERTEX70), scanning electron microscope (SEM, Nova 400 NanoSEM by FEI company, America). Electrochemical workstation (CHI660d), carbon paper, platinum filament electrode (CHI115) and saturated calomel electrode (CHI150) were purchased from CH Instruments Company (Shanghai, China). 

### 2.2. FTIR

The chemical structure of the prepared powder after polymerization was characterized using FTIR spectra (Spectra one, PE Corporation, USA) in the range from 4000 cm^−1^ to 400 cm^−1^ (KBr pellet).

### 2.3. SEM

The surface micromorphology of the PANI powder was characterized by SEM.

### 2.4. Preparation of PANI and PANI Single Electrodes

The 25 mL 1 M HCl and 2.3 mL aniline were successively added to a three-neck flask. The mixed solution was stirred for 1 h under room temperature, and then the oxidant (25 mL, 1 M ammonium persulphate) was dropped slowly into the flask. The mole ratio of ammonium persulphate (APS) to aniline was 1 : 1. After sustaining stir for 6 h, the black-green reactive products were filtered and washed repeatedly with distilled water till the pH value was approximately 6. The PANI powder was then dried in an oven under vacuum at 80°C for 48 h.

The PANI single electrode was made using a mixture of 90 wt% PANI powder (active material), 5 wt% acetylene black (conductive powder), and 5 wt% PTFE binders in ethyl alcohol dispersant to form the slurry with proper viscosity. The slurry of the mixture was then painted onto a carbon paper after weighing which was used as current collector. Then the electrodes were dried in an oven under vacuum at 80°C for 24 h.

### 2.5. Electrochemical Measurement of PANI Single Electrodes

Electrochemical performances of PANI electrodes were measured by charge/discharge technique in the potential window of 0~0.6 V (SCE) and constant current densities of 0.5, 1, 2, 3, 5, and 15 Ag^−1^, respectively, and cyclic voltammetry (CV) with voltage range of −0.4 to 1.1 V (SCE) at various potential scan rates of 10, 50, 100, 200, and 300 mVs^−1^, respectively, on electrochemical workstation. The experiments were carried out using a conventional three-electrode system, which consisted of PANI electrode (working electrode), Pt electrode (counter electrode), and SCE (reference electrode). They were immersed in 1 M HCl or 1 M H_2_SO_4_ electrolyte solution. All experiments were conducted at room temperature.

Consequently, the specific capacitances from galvanostatic charge/discharge curves are calculated using the equation as follows [25]:

(1) $$\begin{array}{c} C_{p}=\frac{I\Delta t}{\left(\Delta V\cdot m\right)}, \end{array}$$

where *C*_*p*_ is the capacitance in Fg^−1^; *I* is the discharge current in ampere (A); Δ*t* denotes the discharge time period in seconds (s); Δ*V* is the potential window in volts (V); *m* indicates the mass of active material in gram (g).

The specific energy can be calculated by [25]

(2) $$\begin{array}{c} E_{p}=\frac{C_{p}U_{\max}^{2}}{2}. \end{array}$$

*E*_*p*_ is the maximum specific energy in Wh·kg^−1^; *U*_max⁡_ is the maximum potential in charge/discharge process, in volts (V).

The coulombic efficiency was calculated by

(3) $$\begin{array}{c} C_{e}=\frac{C_{p}}{C_{pc}}. \end{array}$$

*C*_*e*_ is the coulombic efficiency in percentage (%); *C*_*pc*_ is the specific capacitance in charge process which is calculated using (1), where Δ*t* is the charge time period in seconds.

## 3. Results and Discussion 

### 3.1. FTIR Analysis

**Figure 1:**
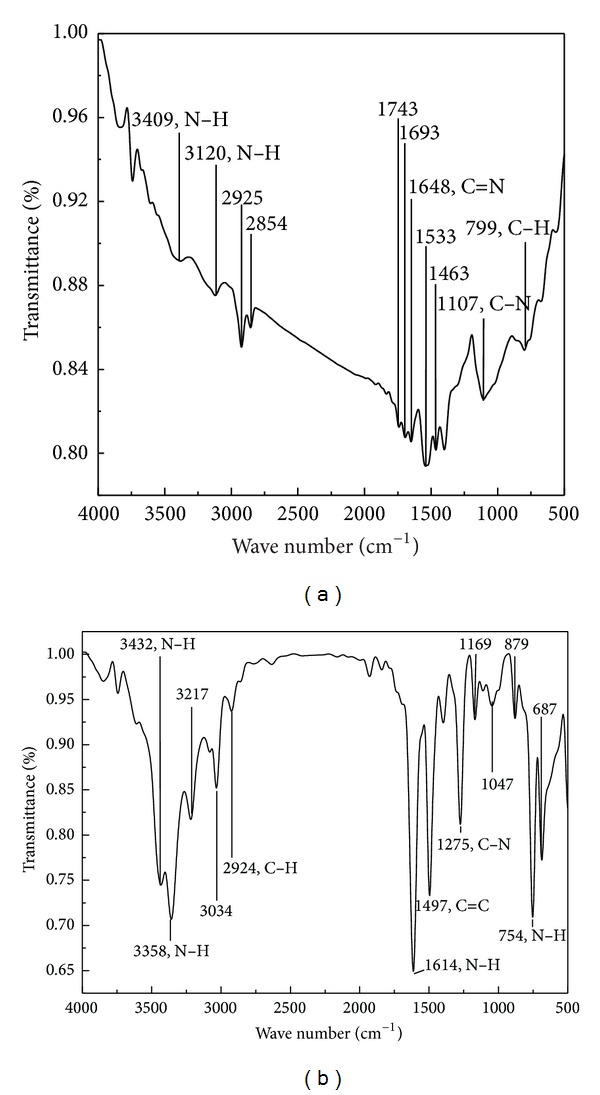
FTIR spectra of PANI (a) and aniline (b).

Figure 1 shows the FTIR spectra of the synthesized powder samples and aniline monomer. Some characteristic peaks in Figure 1(a) are marked, and the corresponding detail implications are assigned as follows: the peaks at 3409 cm^−1^ and 3120 cm^−1^ are attributed to N–H stretching mode. No peaks appeared in the range from 3100 cm^−1^ to 3000 cm^−1^, which indicates that no C–H stretching vibration occurred on benzenoid ring. The absorption peaks observed at 2925 cm^−1^ and 2854 cm^−1^ are due to asymmetric C–H and symmetric C–H stretching vibrations [26]. In addition, there are also several weak peaks that appear in the range of wave numbers from 2000 cm^−1^ to 1600 cm^−1^, which represents the existence of benzenoid ring. The peaks at 1693 cm^−1^ and 1648 cm^−1^ correspond to C=N stretching mode for imine. There are no peaks in the range from 1640 cm^−1^ to 1560 cm^−1^, which means that there is no shear vibration of N–H. The peaks at 1533 cm^−1^ and 1463 cm^−1^ are related to C=C stretching vibration for quinoid and benzenoid rings, respectively. The peak at 1107 cm^−1^ is attributed to C–N stretching mode for benzenoid ring, and the peak at 799 cm^−1^ is assigned to the plane bending vibration of C–H, which is formed during protonation [27, 28]. The characteristic peaks on FTIR spectra of aniline in Figure 1(b) are also marked. The peaks at 3432 cm^−1^ and 3358 cm^−1^ are attributed to N–H stretching vibration, and the approximate peaks can also be observed in Figure 1(a). The peak at 2924 cm^−1^ corresponds to asymmetric C–H stretching vibration, and the same peak position can also be seen in Figure 1(a). The peak at 1614 cm^−1^ represents the N–H bend vibration. The peak at 1497 cm^−1^ is attributed to C=C stretching vibration for benzenoid ring, and the same peak also appeared in Figure 1(a). The peak at 1275 cm^−1^ is related to C–N stretching mode, and the peak at 754 cm^−1^ is assigned to the wag vibration of N–H, which is different in Figure 1(a).

### 3.2. Morphology of PANI

**Figure 2:**
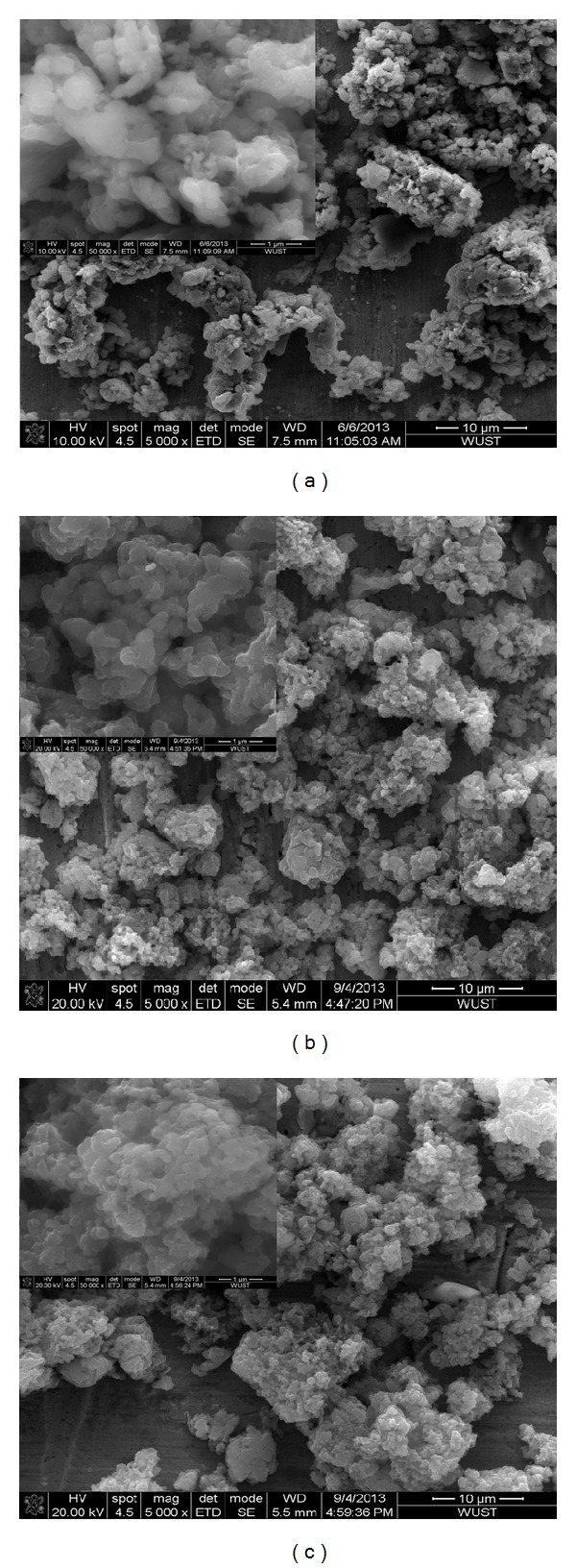
SEM images of PANI ((a) original PANI; (b) PANI immersed in 1 M HCl; (c) PANI immersed in 1 M H_2_SO_4_).

Figure 2 shows the SEM images of PANI by different postprocessing from chemical solution polymerization. The image on the corresponding top left corner is the one in higher magnification. The pictures in Figure 2(a) represent that the original prepared PANI is graininess which looks like globular sponge-like shape indicating that PANI has coarse surface. Figures 2(b) and 2(c) show the morphology of PANI immersed in 1 M HCl and 1 M H_2_SO_4_, respectively. It has been found that the structure of the original PANI and PANI immersed in 1 M HCl and 1 M H_2_SO_4_, respectively, is approximately the same, indicating that immersion of acid electrolyte has no significant influence on the morphology of PANI. This kind of microstructure was considered as having a positive effect on the specific surface area of PANI particles and the diffusion of counteranions [29]; therefore, it is advantageous for the increase of specific capacitance of PANI electrode. However, the rough surface may have negative influence on the conductivity of PANI. Both influences should be taken into consideration in the practical application study of the specific capacitance of PANI.

### 3.3. Electrochemical Performance of PANI

#### 3.3.1. Charge/Discharge Measurement of PANI Electrodes

**Figure 3:**
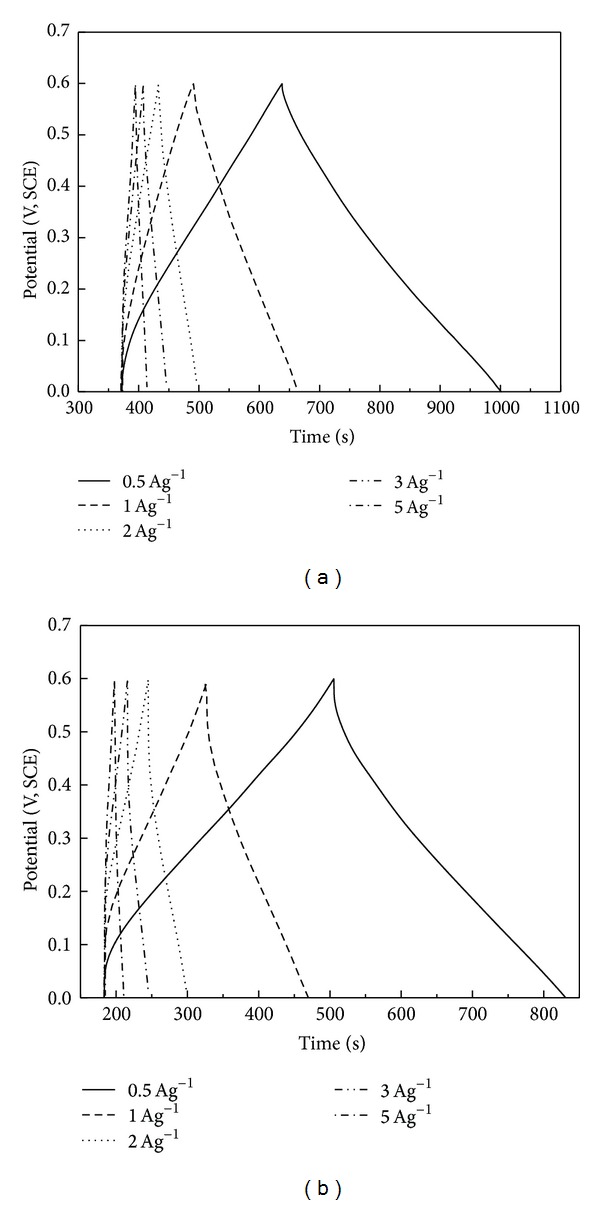
Charge/discharge curves of PANI in 1 M HCl (a) and 1 M H_2_SO_4_ (b) at different current densities of 0.5, 1, 2, 3, 5 Ag^−1^.

**Figure 4:**
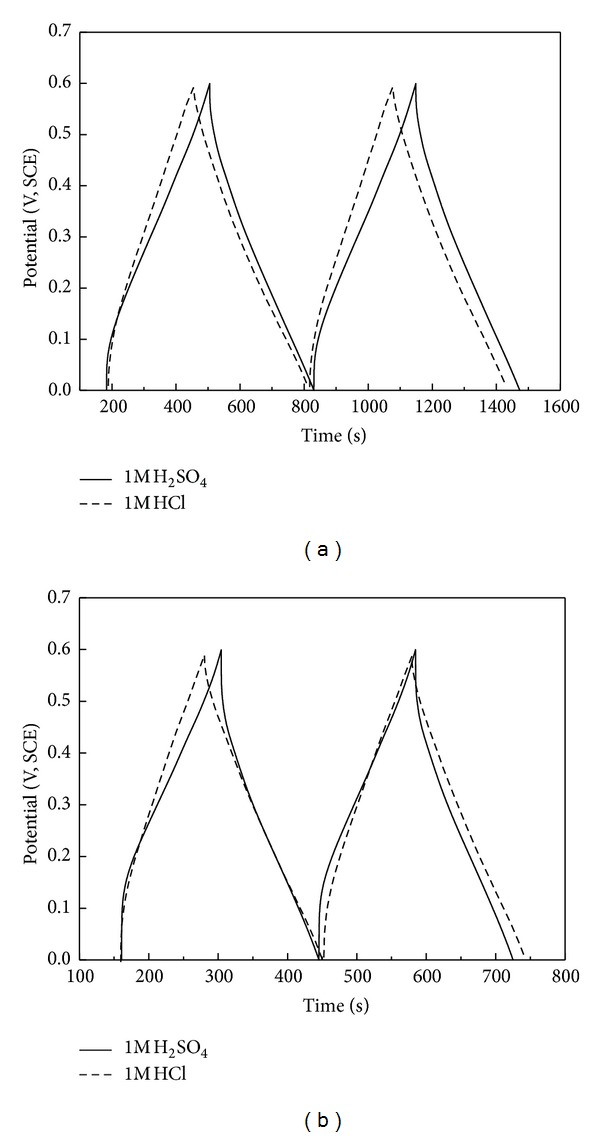
Charge/discharge curves of PANI in different acid electrolytes at the same current density ((a) 0.5 Ag^−1^, (b) 1 Ag^−1^).

Figure 3 shows the charge/discharge curves of PANI in 1 M HCl and 1 M H_2_SO_4_ electrolyte solutions at the current densities of 0.5, 1, 2, 3, and 5 Ag^−1^, respectively. From the plots, good symmetry which represents high coulombic efficiency during charge/discharge process is observed, but the symmetry of plots in 1 M HCl is better than that in 1 M H_2_SO_4_. The IR drops (potential drops) increase apparently with the increase of current density from 0.5 Ag^−1^ to 5 Ag^−1^ in both acid electrolytes shown in Figure 3. To compare the difference of charge/discharge behaviors of PANI in these two kinds of acid electrolytes clearly, the curves at the same current density of 0.5 Ag^−1^ and 1 Ag^−1^ are presented in one plot shown in Figures 4(a) and 4(b), respectively. For the same current density of 0.5 Ag^−1^ or 1 Ag^−1^, the IR drops are more rapid in 1 M H_2_SO_4 _than those in 1 M HCl. It means PANI has better capacitive performance in the latter acid electrolyte.

The values of specific capacitance (*C*_*p*_), specific energy (*E*_*p*_), and coulombic efficiency (*C*_*e*_) of PANI in 1 M HCl and 1 M H_2_SO_4_ solution are calculated using (1), (2), and (3), respectively, and the results are listed in Table 1.

**Figure 5:**
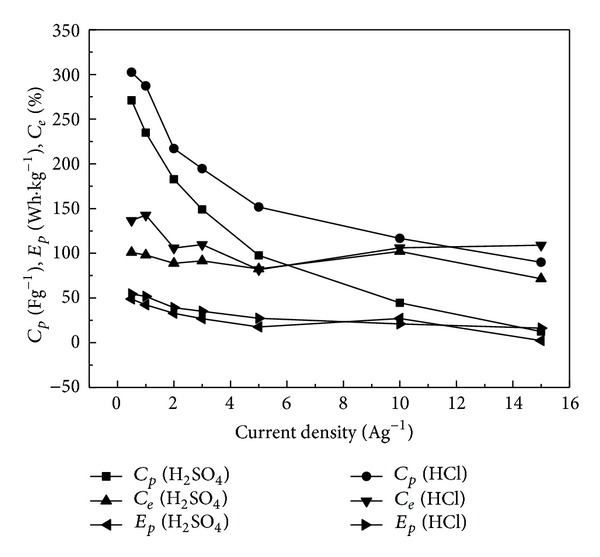
Relationship of specific capacitance (*C*_*p*_), specific energy (*E*_*p*_), and coulombic efficiency (*C*_*e*_) of PANI from charge/discharge curves at current density from 0.5 Ag^−1^ to 15 Ag^−1^ in 1 M HCl and 1 M H_2_SO_4_.

Figure 5 shows that the values of specific capacitance from charge/discharge curves in 1 M HCl electrolyte are higher than those in 1 M H_2_SO_4_, and the higher the current density is, the more remarkable the difference of their specific capacitance is. Thus, it may be inferred that the values of specific capacitance of them may be the same when the current density is low enough, but considering the factor of heavy current discharging, PANI will have better capacitive properties using HCl as electrolyte. Likewise, the corresponding values of coulombic efficiency and specific energy are higher in 1 M HCl solution than those in 1 M H_2_SO_4_. However, the result is opposite at the current density of 10 Ag^−1^, which is caused by the difference of final charge potential, that is, 1.1 V for sulfuric acid and 0.6 V for hydrochloric acid electrolyte. As the value of specific energy can be calculated by (2), it will be more effective by increasing the potential instead of the specific capacitance in order to improve the *E*_*p*_. In fact, the electrochemical response of PANI is related to the species of the concomitant electrolyte negative ion in some extent, and the behaviors in HCl, H_2_SO_4_, and HClO_4_ solutions, respectively, are quite different which are attributed to the different trammels on various kinds of oxidation state of polymer system of different anions [30].

#### 3.3.2. Cyclic Voltammetry Measurement of PANI Electrodes

**Figure 6:**
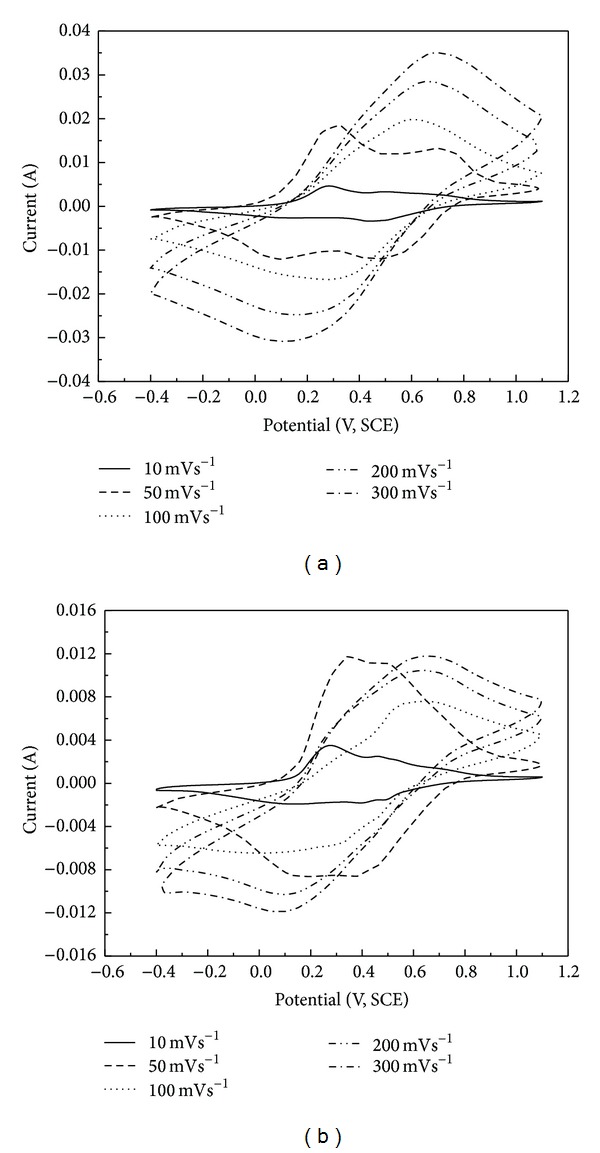
Cyclic voltammograms of PANI in 1 M HCl (a) and 1 M H_2_SO_4_ (b) at different sweep rates of 10, 50, 100, 200, and 300 mVs^−1^.

Figure 6 shows the cyclic voltammograms of PANI in 1 M HCl and 1 M H_2_SO_4_ at the sweep rates of 10, 50, 100, 200, and 300 mVs^−1^, respectively. It shows that two pairs of peaks appeared and are apparent when the sweep rate is low, such as 10 mVs^−1^ and 50 mVs^−1^; but when the potential scan rate increases to 300 mVs^−1^, only one pair of peaks is observed and the peaks became broad thus resulting in the uncertain peaks' potential position. Besides, the potential difference between anodic peak potential and cathodic peak potential becomes bigger because of the higher oxidation peak potential and lower reduction peak potential meanwhile increasing the scanning rate.

**Figure 7:**
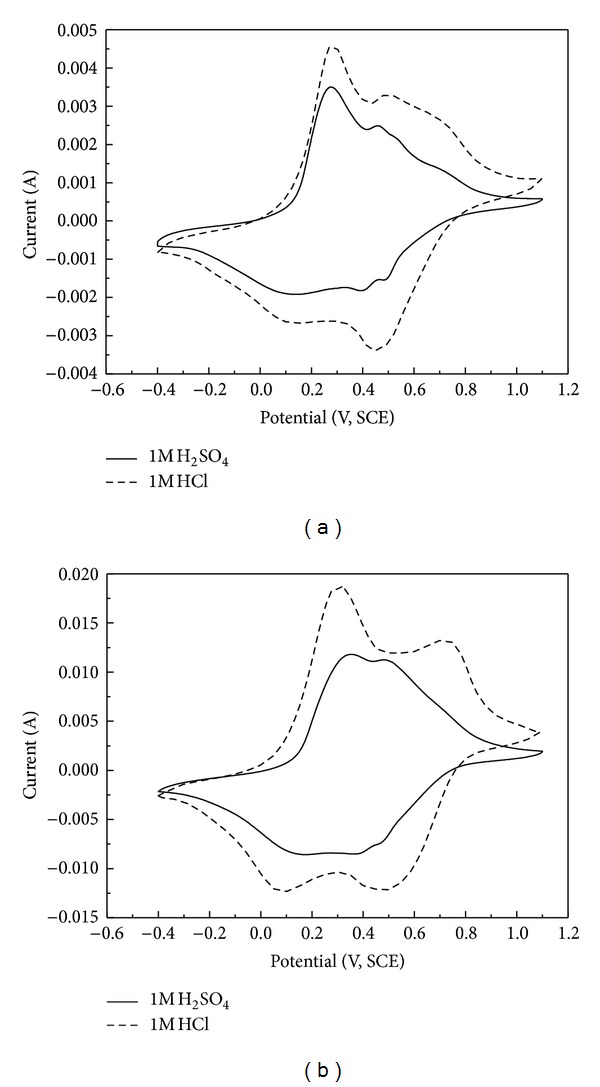
Cyclic voltammograms of PANI in different acid electrolytes at the same potential scan rate ((a) 10 mVs^−1^, (b) 50 mVs^−1^).

Figure 7 represents the steady-state cyclic voltammograms of PANI in 1 M HCl and 1 M H_2_SO_4_ at the potential range of −0.4 V~1.1 V with sweep rates of 10 mVs^−1^ and 50 mVs^−1^. For the same scan rate of 10 mVs^−1^ or 50 mVs^−1^, the peak is more apparent, and the area surrounded by the curve is larger in 1 M HCl than that in 1 M H_2_SO_4_. There are two pairs of redox peaks in both cyclic curves, but the redox peaks are more apparent in the curves obtained in HCl electrolyte. Two approximately reversible oxidation peaks of PANI are observed at 0.72 V and 0.31 V, respectively, in the positive-going scan (forward scan), and the corresponding reduction peaks are also observed at 0.48 V and 0.09 V, respectively, in the negative-going scan in 1 M HCl. The position of the last oxidation potential at 0.72 V is in a good agreement with the literature [10, 17, 18]. For sulphuric acid, it caused smaller peak current but similar peak position at a scan rate of 10 mVs^−1^as shown in Figure 7(a). The PANI electrode is thus seen to exhibit slightly different cyclic voltammetric behavior in different acid electrolytes which may be caused by the different size and charge of acid. Further research of the influence of counter ion on electrochemical behavior of PANI has been done in the literature [18], which proves to be that the influence of anion charge on counter anion is more important than that of size, and SO_4_^2−^ is in higher charge and bigger size than Cl^−^. This indicates that anion migration close to the electrode, that is, anion mobility, is an important factor for the electrochemical nature of PANI electrode [18].

The comparison of the first pair of redox peak of the two curves shows that the redox peak potential in HCl electrolyte is smaller than the corresponding potential in H_2_SO_4_, but the opposite result is shown in the second pair of peak, which indicates that the working potential of PANI single electrode using HCl as an electrolyte is higher than that using H_2_SO_4_. When the oxidation potential and reduction potential are the same or similar in the same pair of redox peak, better symmetry of cyclic voltammograms can be observed which means that the cyclic reversibility of the PANI electrode is better, and the symmetry of the corresponding charge/discharge curves is more apparent. The bigger the potential difference between the first pair redox peak and the second one is, the higher the working potential of the capacitor using the matter as active material is. Because of faradic process, the storage of charge lies on the migration of electron accompanying the oxidation state change of electrode active material which complies with Faraday's Law and relates to electrode potential. Faradaic pseudocapacitance will arise if the two dimensional or quasi-two dimensional faradic reaction occur in special situation. The energy storing mechanism is similar to the batteries [30].

**Figure 8:**
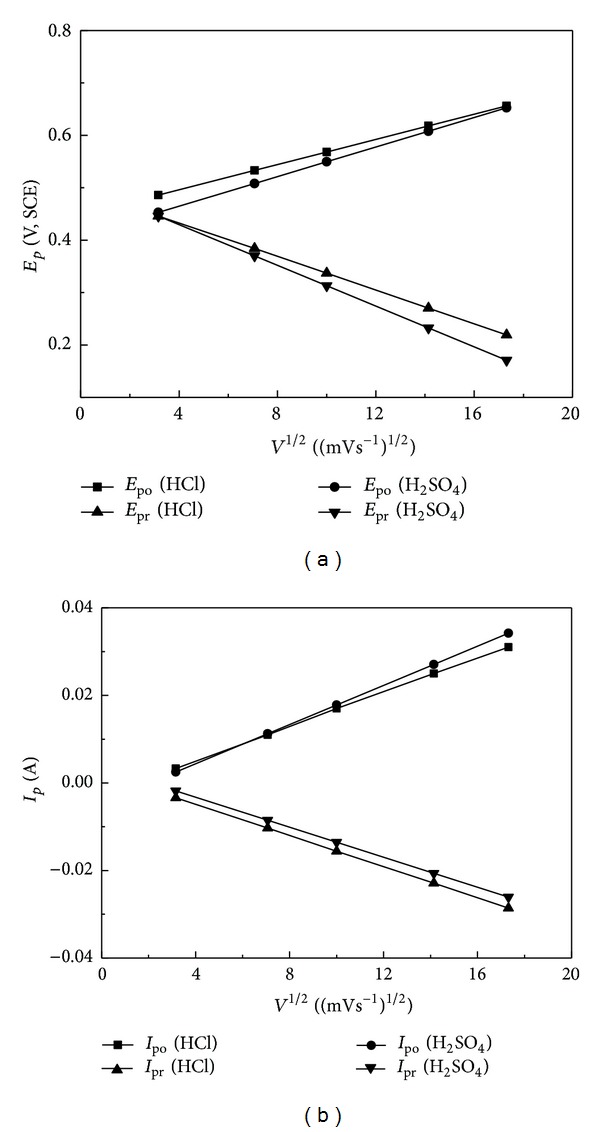
Relationship graphs of (a) peak potential, *E*_*p*_, and (b) peak current, *I*_*p*_, for oxidation and reduction from cyclic voltammograms in Figure 6 to square root of scan rates in 1 M HCl and 1 M H_2_SO_4_.

The relationship between the anodic and cathodic peak potentials or peak currents and the square root of the scan rate from 10 mVs^−1^ to 300 mVs^−1^ is shown in Figure 8. The linear relation of each curve can be gotten, but the slope of each curve is different. These results indicate that diffusion of species which may be from electrode or solution to the electrode surface is the determining step, but any differences observed between different acid electrolytes are likely to be due to the negative counter ion. The variation of peak potentials may thus reflect the difference of diffusion rate for oxidation and reduction. The slight variation of the slopes relating to the oxidative peaks between hydrochloric acid and sulfuric acid can also be explained by the difference of size and charge of counter ion.

## 4. Conclusion

PANI with globular sponge-like morphology is prepared successfully by chemical polymerization. The charge/discharge behaviors of PANI in 1 M HCl and 1 M H_2_SO_4_ are different. For the same current density of 0.5 Ag^−1^ and 1 Ag^−1^, PANI has better capacitive performance in 1 M HCl than that in 1 M H_2_SO_4_. The values of specific capacitance, as well as specific energy and coulombic efficiency of PANI in 1 M HCl, are higher than that in 1 M H_2_SO_4_ at the same current density. For cyclic voltammograms behavior, two pairs of peaks are apparent at low sweep rates of 10 mVs^−1^ and 50 mVs^−1^. Moreover, the potential difference between anodic peak and cathodic peak becomes heavier with the increase of scan rate. The diffusion of species to the electrode surface from electrode or solution is the determining step in redox process. The variation of peak potentials reflects that the diffusion rate for oxidation and reduction is different. However, for further explanation of the reasons, more and further researches on the influence of pH and concentration of one electrolyte or other several kinds of acid electrolytes should be done.

## Figures and Tables

**Table 1:** The values of specific capacitance (*C*_*p*_), specific energy (*E*_*p*_), and coulombic efficiency (*C*_*e*_) of PANI from charge/discharge curves at current density from 0.5 Ag^−1^ to 15 Ag^−1^ in 1 M HCl and 1 M H_2_SO_4_.

Current density (Ag^−1^)	*C*_*p*_ (Fg^−1^)		*E*_*p*_ (Wh·kg^−1^)		*C*_*e*_ (%)	
	H_2_SO_4_	HCl	H_2_SO_4_	HCl	H_2_SO_4_	HCl
0.5	270.85	302.43	48.75	54.44	100.9	136.6
1	234.68	287.02	42.24	51.66	98.0	142.8
2	182.66	217.00	32.88	39.06	88.8	105.9
3	149.00	194.50	26.82	35.01	91.4	109.6
5	97.50	151.67	17.55	27.30	82.4	81.6
10	44.55	116.67	26.95	21.00	102.0	106.1
15	12.50	90.00	2.25	16.20	71.4	109.1
